# Impact of Leadership and Mobility on Consensus-Building in Sensor Networks

**DOI:** 10.3390/s20041081

**Published:** 2020-02-17

**Authors:** Roya Norouzi Kandalan, Murali Varanasi, Bill Buckles, Kamesh Namuduri

**Affiliations:** Department of Electrical Engineering, University of North Texas, Discovery Park, 3940 N Elm St, Denton, TX 76207, USA; Murali.Varanasi@unt.edu (M.V.); Bill.Buckles@unt.edu (B.B.); Kamesh.Namuduri@unt.edu (K.N.)

**Keywords:** consensus-building, sensor models, sensor network, Vehicle-to-Vehicle (V2V) Communications, mobility

## Abstract

Introducing leadership and mobility is known to benefit wireless sensor networks in terms of consensus-building and collective decision-making. However, these benefits are neither analytically proven nor quantified in the literature. This paper fills this gap by investigating the mobility dynamics in wireless sensor networks analytically. The results of the analytical investigation are presented as a set of theorems and their proofs. This paper also establishes a natural synergy between the leader-follower model and its bipartite graph representation. It demonstrates the advantages of the leader-follower model for consensus-building over others in terms of improved convergence rate. It presents a strategy for choosing leaders from among the agents participating in the consensus-building process using the well-known graph-coloring solution. Then, it shows how the leader-follower model helps improve the convergence rate of consensus-building. Finally, it shows that the convergence rate of the consensus-building process can be further improved by making the leaders mobile.

## 1. Introduction

Communication, coordination, and decision-making represent the three main components of an intelligent transportation system. Vehicles communicate with one another using V2V and/or to other infrastructure using vehicle-infrastructure (V2I) communications. Coordination happens through information exchange among vehicles. Decision-making requires algorithms such as consensus-building. Consensus-building is an iterative process that results in a collective agreement among the participating agents. Consensus-building has been applied to solve problems such as distributed estimation and motion coordination [1] in many fields, including intelligent transportation.

Consensus-building refers to collective decision-making or estimation of the value of a parameter in a distributed network. The decision or the final consensus value of this parameter depends on the opinions of all members of the group. The process of reaching a consensus is primarily built upon the interactions among agents and their connected neighbors. Consensus-building or collective decision-making plays an essential role in the daily lives of humans as well as animals [2]. Event planning and election process are simple examples of collective decision-making. In sociology, consensus-building is known as opinion formation.

### 1.1. Motivation

Consensus-building in distributed systems has been investigated thoroughly in the literature. Further, it is generally known that introducing leadership and mobility improves the rate of consensus-building. However, there are no analytical proofs in the literature that clearly demonstrate the benefit of introducing leadership and mobility into distributed Multi-Agent-Systems (MAS) in the literature to date. This motivated us to investigate analytical strategies for demonstrating the advantages of leadership and mobility and methods to achieve faster convergence rates in consensus-building.

### 1.2. Comparison with Existing Works

In the existing literature, consensus-building has been studied within the context of unipartite network typology. In our previous research [3,4], we first introduced bipartite topology to represent leader-follower model and investigated the convergence characteristics of consensus-building processes. However, our previous research did not include any analytical proof or demonstration of the advantages of leadership or mobility. This (current) paper primarily focuses on filling this gap. Here, we demonstrate the advantage of leadership and mobility in consensus-building by proving three theorems. Further, we develop a method to convert a unipartite graph into a bipartite graph using the well-known node-coloring algorithm and a strategy to choose the leader subset. As opposed to classical leader selection strategies, in leader-follower model, leader selection refers to identifying one of the two-partitions as the leader subset. The leader-follower scheme developed in this work reduces the complexity of mobility analysis. It allows us to investigate the advantages of having mobile leaders better.

### 1.3. Contributions of This Paper

This paper contributes to the state of the art in consensus-building in two dimensions. First, it investigates the leader-follower model with a bipartite topology and the importance of leader selection in improving the convergence rate of consensus-building in a distributed system. It proposes bipartite graph as a suitable representation for the leader-follower model and brings the synergy between the two. It presents a leader selection process based on the graph-coloring scheme. The transition matrix corresponding to the convergence of the consensus-building process is derived for the leader-follower model. It suggests that the final consensus value obtained from the consensus-building is a weighted consensus where the weights are determined by the number of connections for each agent. The second major contribution of this paper is an analysis of the impact of mobility on consensus-building. It suggests that by making the leaders mobile, the convergence rate of consensus-building can be improved.

### 1.4. Organization

A brief survey of relevant literature is presented in Section 2. Consensus-building in leader-follower model is discussed in Section 3. Mobility analysis in leader-follower model is discussed in Section 4. Summary and conclusions are presented in Section 5.

## 2. Background and Literature Survey

Collaborative decision-making or consensus-building has widespread applications in many branches of knowledge and science, such as autonomous computing, wireless sensor networks, and robotics. It even has interesting applications in data mining and clustering [5]. The importance of consensus-building in modern intelligent transportation systems is very explicit. V2V and V2X communications help disseminate information about accidents, congestion, and potential obstacles. V2V and V2X communications and its application are extensively analyzed in References [6,7].

Autonomous vehicles require communication and coordination with their neighbors. Methods to maintain reliable communications among vehicles through a peer to peer communication have been explored in Reference [1]. Applications of consensus-building in autonomous vehicles include task management [8], formation control [9] and intersection management [10].

Network topology significantly impacts communication in a vehicular network. Ruan et al. [11] explored the impact a leader-follower topology has on a network with a limited range of communication. When studying a network topology, one of the major challenges in reaching an agreement is when nodes fail. Faulty nodes caused when a vehicle, sensor, or agent in the network has depleted battery power or provides misleading results. Efficient and fast convergence in a network can be achieved with the right choice of consensus algorithm [12].

A dynamic model to explain consensus-building in groups of individuals is proposed in Reference [13]. Decision-making in ant colonies demonstrating the impact of “wisdom of the crowds” is investigated in Reference [14]. In bird-flocking, the impact of a pair of birds on the consensus-building is proportional to their distance [15]. Consensus-building plays a vital role in environmental science as well as in physical science. Load scheduling for power systems using consensus-building is studied in Reference [16]. From a control-theory perspective, collective decision-making has applications in robotics, sensor networks, autonomous vehicles, and unmanned aircraft systems. Consensus-building among a group of individuals has been a topic of interest in management science and statistics since 1960.

Consensus-building using subjective probability distribution is explained in Reference [17]. Olfati Saber et al. studied linear and non-linear consensus-building in directed and undirected graph topologies. In their work, time delay, node/link failure, and the robustness of network topology to variations were investigated. In their model, the Perron matrix was used as a transition matrix and, Fiedler eigenvalue was used as a measure for reaching consensus [18,19,20]. Vicsek’s [21] proposed a model to describe the emergence of collective motion in swarming systems. This model studies the behavior of particles to their neighbors. It suggests that the direction of the motion of each particle is proportional to the average of its neighbors’ direction. It also studies the impact of noise in cohesive motion. Later on, Jadbabai [22] used this model to study the nearest neighbor motion interactions. They used Vicsek’s model to prove that the nearest neighbor rule leads agents to move in a particular direction.

On the other hand, Boyd et al. in Reference [23] considered random walk model and built the transition matrix based on edge weight. Their work concentrates on the optimization of the second largest eigenvalue modulus (SLEM) of the system. The SLEM is an indicator of the algebraic connectivity of a graph that represents a multi-agent system. Algebraic connectivity of a graph, in turn, indicates the rate of convergence of the distributed system the graph represents. Hoshaverdi et al. explored consensus-building in small-world topologies [24,25]. They also proposed the idea of selective leaders in a two-level hierarchical graph, which helps to reach consensus faster.

Building on their previous work [3], Norouzi et al. in Reference [4] investigated the impact of the bipartite graph topology on consensus-building. They also proposed the leader selection strategy that minimizes the number of iterations required to reach consensus. Ren et al. investigated consensus-building in dynamic graphs [26]. Their work proves that if the union of graphs in each state has a spanning tree, the whole system can reach consensus in a finite number of iterations. Tahbaz-Salehi et al. proved that consensus-building could be simplified to the problem of weak ergodicity in random graphs [27].

## 3. Consensus in a Leader-Follower Model

Graph representation is suitable for modeling the interactions among agents in a distributed network. Let G=(V,E) represent a graph with a set of nodes V={1,...,V}, connected through edges E⊆V×V.

Graphs are general representations of a network. The edges of a graph represent the connections among the nodes. In the next section, the principles of leader-follower model will be explained. The topology of a network and its connections will be explained. Further, the matrices associated with a network will be generated and analyzed.

### 3.1. Consensus in Leader-Follower Model: Results and Discussion

The leader-follower model, as a two-level hierarchical structure, has been examined extensively in the literature. The nodes in this structure are labeled as leaders or followers. In our work, the main focus is the particular case of leader-follower’s model, in which connections among the peers of a set is prohibited. This assumption, which is the core in this work, demands the elimination of any connection between any two leaders or any two followers.

According to this argument, if the set of all of the leader vertices is represented by L={1,...,l} and the set of all of the followers is demonstrated by F={1,...,f}, an edge between any two nodes in a leader-follower model exists only if one is from L, and the other is from F, as shown in Equation (1).

(1) (i,j)∈E if [i,j]⊄L & [i,j]⊄F.

In Equation (1), *i* and *j* represent the indices of nodes. Two nodes are connected if and only if one belongs to the set of leaders, and the other belongs to the set of followers. It should be noted that a node *v* is either a leader or a follower. In other words, the union of the two sets is the set of all the vertices (See Equation (2)). Equation (3) implies that a node cannot be a member of both sets.

(2) L∪F=V

(3) L∩F=∅.

A modified bipartite graph is a suitable representation for the nodes following Equation (1). A bipartite graph naturally represents a two-level hierarchical model. In general, a bipartite graph is a graph whose nodes can be partitioned into two disjoint sets. Connections exist only between members of different groups. There is no connection among the peers (members of the same group). In a bipartite graph, self-loops are banned. In the modified version of the bipartite graph used in this work, self-loops are allowed, as represented in Equation (4).

(4) (i,i)∈E.

Self-loops indicate that every agent includes its own opinion in reaching a consensus. The number of iterations that a network takes to reach consensus is a function of its degree distribution.

### 3.2. Bipartite Representation

The necessary and sufficient condition to represent a graph in a bipartite form is to have no odd cycle in the graph. An odd cycle is a chain made of an odd number of links, which starts and terminates at the same node. In order to apply the principle of the leader-follower model to more extensive classes of graphs, we need a strategy to eliminate odd cycles from a given graph. One approach to remove odd cycles is through the node coloring technique. This technique starts with an arbitrary node that is colored in red. In the following step, all its neighbors are colored in black. This process is continued until the last node is colored. Finally, the links, which are connecting the same color nodes, will be excluded.

In order to optimize the process of removing odd cycles and to minimize the number of eliminated links, the maximum-cut approximating scheme can be adopted. The purpose of this method is to erase the minimum number of links to convert a planar graph to a bipartite form. To begin with, assume that all nodes are colored in red. In this situation, the number of edges connecting red nodes to black nodes is zero. Starting with an arbitrary node *S* and coloring it in black, the number of edges connecting the red nodes to the black nodes increases for each of the connections of the node *S*. As a result, if node *S* has $e_{s}$ connections, the size of the cut increases by $e_{s}$. In the next step, a node $S^{\prime }$, which is node *S*’s neighbor, will be colored in black. In this case, the number of links connecting the black nodes to the red nodes will be increased by $e_{S^{\prime }}-1$. The link connecting the node *S* to the node $S^{\prime }$ is not a connecting link between the red nodes and the black nodes.

The process of switching node’s color continues until there is no such node that has more connections with its peers than it has with the other color group. The algorithm may involve back and forth color switch for each node. This algorithm starts with a zero number of links connecting the red group to the black group as the initial condition was to have all the nodes in the red color. On the other hand, if the graph has no odd cycle, there is a unique bipartite representation for the graph with *e* edges connecting the red group to the black group. Consequently, as the algorithm starts with a value of 0 and gets increased to the maximum value of *e*, it must terminate. The running time for the algorithm is in the order of O(e(e+n)). Goemans and Williamson, in Reference [28], proposed a randomized approximation algorithm to enhance the solution delivery time to 0.87856 times its optimal value. This algorithm is summarized in Algorithm 1.
**Algorithm 1:** Node coloring algorithm to convert unipartite graph into bipartite graphred=Vblack=∅e=0% Number of edges connecting nodes in set red to the nodes in set black$\bar{e}=E$% Number of edges connecting nodes in the same setChoosearandomnodevSwitch(v)% Switch()changes the color of the node *v*e=0+deg(v)**while***∃(v∈V) for which Switch (v) increases e;***do**| Switch(v)**end****if***$\bar{e}\neq 0$***then**| cut all the edges in $\bar{e}$**end**

### 3.3. Leader Selection Process

After transforming the graph into its bipartite representation, one of the two partitions must be labeled as leaders and the other as followers. Previous studies on leader selection provide a reference point to select leaders wisely. Intuitively, the size of the leader group has to be smaller than the size of the follower group. Besides, the degree distribution and the number of leaf nodes in each group have significance in the leader selection. The group, which includes a lower number of leaf nodes, serves better as a leader group. However, if both groups (partitions) happen to have leaf nodes with different chain lengths, the group whose leaf nodes are closer to the core of the structure can be considered more suitable as leaders. As observed in Reference [4], if the proper partition were chosen as the set of leaders, fewer iterations are required to reach consensus among the agents.

### 3.4. Transition Matrix

A transition matrix defines the relation of the states of the network to one another. In our work, a discrete-time Markov chain representing the network has been defined to study the long-run behavior of the system. Suppose the state of the network at time *n* be denoted by $X_{n}$. The initial state of every individual node v∈V is represented by a column vector, as shown in $X_{0}$ Equation (5).

(5) $$X_{0}={\left[x_{1}\left(0\right),x_{2}\left(0\right),...,x_{V}\left(0\right)\right]}^{T}.$$

In Equation (5), $X_{0}$ holds the initial values of all the individual nodes. For instance $x_{1}\left(0\right)$ represents the initial value of node one.

Every transition alters the values of the nodes and consequently modifies the state of the network. Transition from the state at time *n* to the state at time n+1 is described by Equation (6):

(6) $$P(X_{n+1}=i_{n+1}|X_{n}=i_{n},X_{n-1}=i_{n-1},...,,X_{0}=i_{0}).$$

Equation (6) reflects the transition in a system with memory. In this equation, $X_{n+1}$ = $i_{n+1}$ represents the state of the system at time n+1 which in turn, depends on all its previous states from $X_{0}$ = $i_{0}$ to $X_{n}$ = $i_{n}$. To be more specific, $i_{n}$ represents the value of the state $X_{n}$. As mentioned earlier, the leader-follower model in this work has *Markov property*. As a result, Equation (6) is simplified to Equation (7) [29]:

(7) $$p_{ij}=P\left(X_{n+1}=i_{n+1}|X_{n}=i_{n}\right).$$

Each state only depends on its immediate previous state, as indicated by Equation (8).

(8) $$X_{n+1}=PX_{n}.$$

The transition matrix, P, is a projection of the network topology and its connectivity. Transition matrix P is a non-negative matrix as shown in Equation (9).

(9) $$p_{ij}\geq 0.$$

The column sum of every row is equal to one, as shown in Equation (10).

(10) P1=1.

In Equation (10), 1 represents a column matrix of all ones, ${\left[1,1,...1\right]}_{1\times n}^{T}$.

The network of interest is established based on connections to the leader. It is assumed that if two nodes share at least one leader as shown in Equation (11), then their corresponding element in the transition matrix will be non-zero, as shown in Equation (12).

(11) [i,j]⊆F & [k]⊆L,

(12) $$\left(i,k\right)\in E\;\&\;\left(j,k\right)\in E\to p_{ij}\neq 0.$$

Equations (11) and (12) imply that if two nodes *i* and *j* have at least one leader in common, the value of the the element $p_{ij}$ in the matrix is not zero. It should be noted that if node *i* and node *j* are from the same partition, as per the rules of a bipartite graph, there is no connecting edge, as shown in Equation (13).

(13) $$p_{ij}\neq 0↛\left(i,j\right)\in E.$$

The strength of the connection in this setting is defined by Equation (14).

(14) $$p_{ij}=\frac{1}{N}\sum_{c=1}^{M}\frac{1}{Deg(c)+1}.$$

In Equation (14), *i* and *j* are two distinct followers, *N* is the total number of leaders to which *i*th sensor is connected, *M* is the number of leaders connecting the sensors *i* and *j*, and Deg(c) is the degree of *c*th leader.

Let us consider a scenario in which *i* is a leader, and *j* is its follower. In this scenario, *N* is one, because *i* has no other leader in common with follower *j*. On the other hand, suppose *i* is the follower, and *j* is one of its leaders. In this case, *N* would be the degree of the node *i*, but the summation would be limited to only leader *j*, because, *j* does not have any common follower with *i*. Finally, if both *i* and *j* are leaders, the value of $p_{ij}$ will be zero because there is no mutual leader between any two leaders.

Intuitively, Equation (14) suggests that the followers connected with more number of the leaders have more influence on the final agreement in the network.

The main focus of this work is the study of long-term behavior of the network in reaching an agreement among the agents regardless of their label. An agreement is reached when the system achieves stability and remains unchanged, as indicated in Equation (15).

(15) $$P^{n+1}=P^{n}.$$

The number of required iterations, *n*, is estimated using Equation (15). The transition matrix for the leader-follower model is generated using Equation (14). Before moving on to the long-term behavior of this network, an example is provided to illustrate the transition matrix.

**Figure 1:**
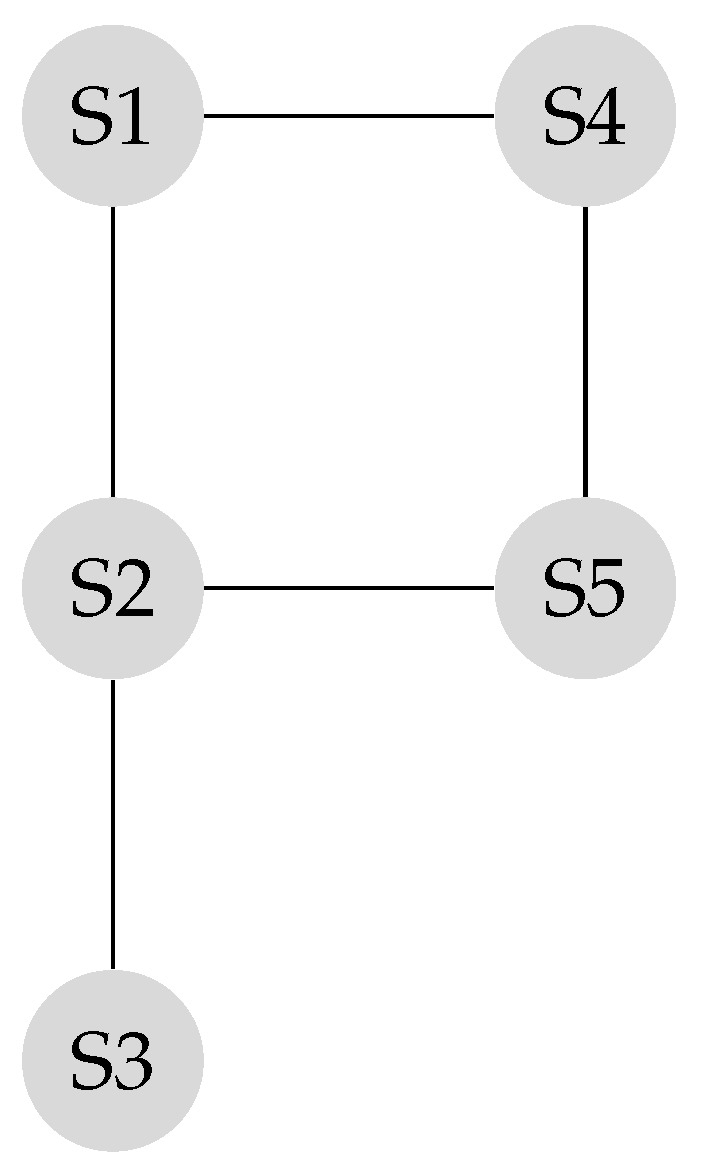
Original unipartite graph.

**Figure 2:**
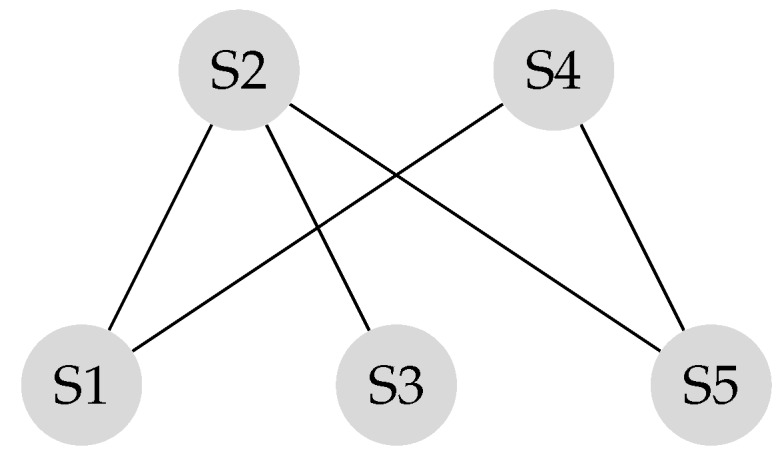
Bipartite graph corresponding to Figure 1.

**Figure 3:**
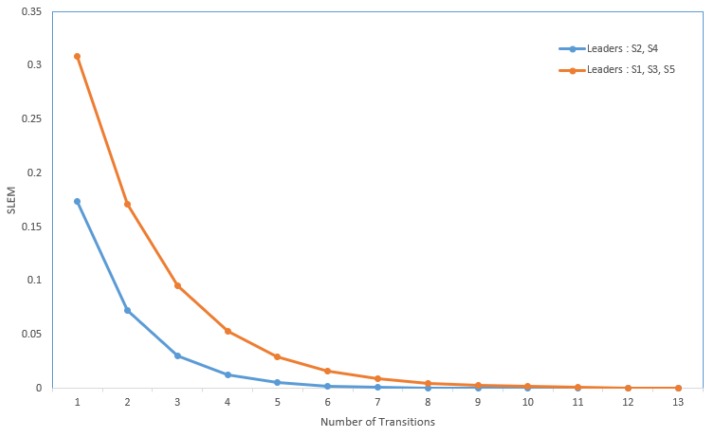
Improvement in convergence rate due to suitable selection of leader subset.

Suppose the graph shown in Figure 1 has to be converted to a leader-follower model. Figure 2 demonstrates a bipartite representation generated from the unipartite graph shown in Figure 1 using the leader-follower model. Self-loops in the bipartite graph are not depicted. The assumption is that every node holds onto its opinion in every iteration. The leader selection procedure, which is explained in detail in our previous work [4], has been used to compare the long-term behavior of the network with different choices of leaders. Figure 3 compares the SLEM of this network with two different choices of partitions as leaders. In this illustration, selecting {S2,S4} as leaders based on the strategy outlined in Reference [4] lowers the number of iterations required to reach a common agreement among the agents in comparison with the alternative in which {S1,S3,S5} are selected as leaders.

The transition matrix *P* corresponding to the bipartite graph shown in Figure 2 is generated using Equation (14).

$$P=\left[\begin{array}{ccccc} \frac{7}{24} & \frac{1}{8} & \frac{1}{8} & \frac{1}{6} & \frac{7}{24}\\ \frac{1}{4} & \frac{1}{4} & \frac{1}{4} & 0 & \frac{1}{4}\\ \frac{1}{4} & \frac{1}{4} & \frac{1}{4} & 0 & \frac{1}{4}\\ \frac{1}{3} & 0 & 0 & \frac{1}{3} & \frac{1}{3}\\ \frac{7}{24} & \frac{1}{8} & \frac{1}{8} & \frac{1}{6} & \frac{7}{24} \end{array}\right]$$

### 3.5. Convergence to Weighted Consensus

Before starting the discussion on the speed of convergence, it is necessary to outline the stationary status of this network. In this work, we use weighted consensus, that is, it is closer to the opinion of the agent who is connected to a greater number of leaders.

The transition matrix, which is generated using Equation (14), assigns a weight to each agent’s opinion. As a result, the number of neighbors each agent has, as well as the number of the leaders that are connected to each agent, affect the consensus value. The final agreement among the agents, or the stationary state of the network, ($X_{s}$) can be estimated using Equation (16).

(16) $$X_{s}=\frac{\left[N(1),N(2),\ldots N(i),\ldots ,N(K)\right]}{\sum_{j=1}^{M}\left(Deg\left(j\right)+1\right)}X\left[0\right].$$

In Equation (16), *K* is the number of agents, *M* is the number of leaders, N(i) is the number of leaders sensor *i* is connected to, and X[0] represents the initial state of the agents.

For illustration, the stationary state of the network represented in Figure 2 is computed using Equation (17) as follows:

(17) $$X_{s}=\frac{\left[\begin{array}{ccccc} 2 & 1 & 1 & 1 & 2 \end{array}\right]}{7}X\left[0\right].$$

In this approach, the final consensus value is a weighted average of the agents’ initial values. Agents with more connections pull the final value towards their opinion.

So far, the nuances of the leader-follower model of interest have been presented, thus providing the necessary background for investigating the long-term behavior of the network. The next section presents the convergence of a leader-follower model to its stationary state.

### 3.6. Rate of Convergence to a Consensus

A network reaches consensus when all nodes agree upon a value for a given parameter of interest. When the network reaches its stationary state, a consensus is achieved (See Equation (15)). The rate of convergence to a consensus in a leader-follower structure depends on topology and connectivity of the network. The transition matrix is shown in Equation (14) represents the connectivity of the network illustrated in Figure 2. The elements of the transition matrix are, non-negative and the summation of all columns of every row in the transition matrix is one.

**Communication Classes**: Consensus-building in a network depends on the number of communication classes in that network. If there are multiple communication classes, then information is shared within each communication class, but not with other classes. If there is only one communication class in the network, then all agents in the network will be able to share their opinions with others in a few transitions. A network with only one communication class is called irreducible. In other words, the network and its corresponding transition matrix *P* are irreducible when there is a path between every pair of agents in the network.

**Aperiodic Graph**: The leader-follower network has a modified bipartite representation. For a purely bipartite graph, the greatest common divisor (GCD) of lengths of all the paths from one agent to itself is at least two. In the modified bipartite representation, every agent has a self-loop to itself. The self-loop decreases the GCD to one. A network is aperiodic if the GCD of the lengths of all paths which connect an agent to itself is one.

According to the Perron-Frobenius theorem, the mixing rate of an irreducible and aperiodic network is proportional to the Second Largest Eigen Modulus (SLEM) of the transition matrix associated with that network ( See Equation (18)) [30].

(18) $$P^{n}=1\pi^{T}+O(n^{m_{2}-1}|\lambda_{2}{|}^{2}).$$

In Equation (18), π is the right eigenvector of transition matrix *P* associated with the eigen value one. Given that the transition matrix is stochastic, its largest eigenvalue equals one. According to the Perron-Frobenius theorem, the largest eigenvalue of the transition matrix is strictly greater than the magnitudes of all other eigenvalues of the transition matrix, as indicated in (19).

(19) $$\lambda_{1}>|\lambda_{2}\left|\geq ...\geq \right|\lambda_{r}|.$$

Equation (19) indicates that the SLEM of any transition matrix is a number that is strictly less than one. The magnitude of $\lambda_{2}$ is inversely proportional to the rate of convergence in the leader-follower network.

In the next section, the transition matrix of the network with multiple classes of communication will be studied. The impact of mobility in accelerating the rate of convergence in a network of disconnected agents is discussed.

## 4. Mobility in Leader-Follower Model: Results and Discussion

The transition matrix *P*, also referred to as the system matrix, defines the convergence of the system to the weighted consensus, as discussed in the previous section. This matrix is helpful in modeling and analysis of mobility scenarios. Mobility models such as the “Round Robin” alter the transition matrix in each state, and the final transition matrix is equivalent to the product of the instantaneous transition matrices. Assume the locations of the S2 and the S4 in Figure 2 switch periodically.

**Figure 4:**
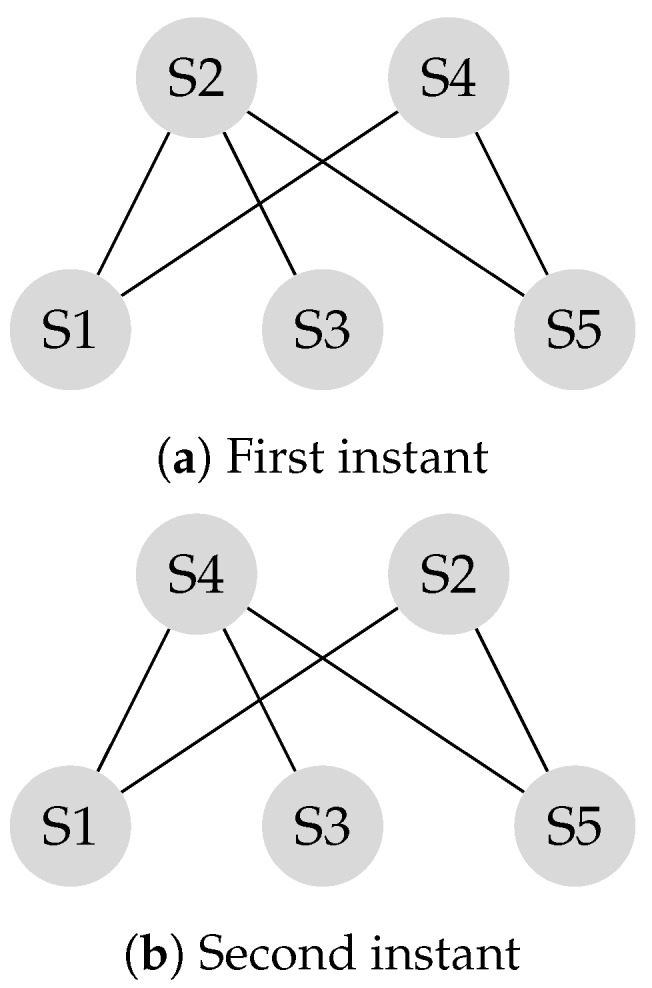
First instant

$P_{1}$ is the transition matrix corresponding to the first instant shown in Figure 4a and $P_{2}$ is the transition matrix corresponding to the second instant shown in Figure 4b.

$$P_{1}=\left[\begin{array}{ccccc} \frac{7}{24} & \frac{1}{8} & \frac{1}{8} & \frac{1}{6} & \frac{7}{24}\\ \frac{1}{4} & \frac{1}{4} & \frac{1}{4} & 0 & \frac{1}{4}\\ \frac{1}{4} & \frac{1}{4} & \frac{1}{4} & 0 & \frac{1}{4}\\ \frac{1}{3} & 0 & 0 & \frac{1}{3} & \frac{1}{3}\\ \frac{7}{24} & \frac{1}{8} & \frac{1}{8} & \frac{1}{6} & \frac{7}{24} \end{array}\right]$$

$$P_{2}=\left[\begin{array}{ccccc} \frac{7}{24} & \frac{1}{6} & \frac{1}{8} & \frac{1}{8} & \frac{7}{24}\\ \frac{1}{3} & \frac{1}{3} & 0 & 0 & \frac{1}{3}\\ \frac{1}{4} & 0 & \frac{1}{4} & \frac{1}{4} & \frac{1}{4}\\ \frac{1}{4} & 0 & \frac{1}{4} & \frac{1}{4} & \frac{1}{4}\\ \frac{7}{24} & \frac{1}{6} & \frac{1}{8} & \frac{1}{8} & \frac{7}{24} \end{array}\right]$$

**Figure 5:**
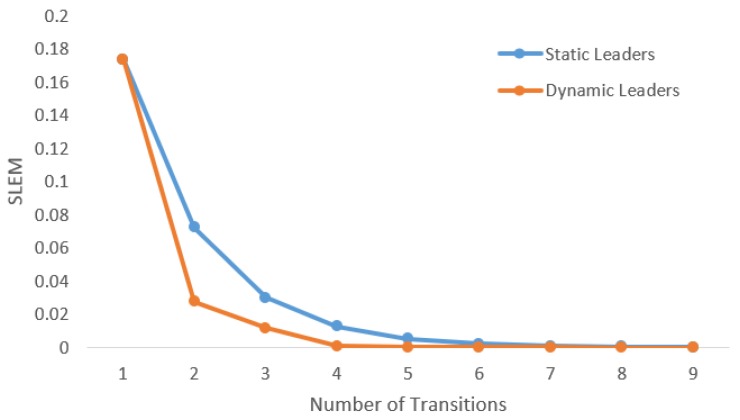
Improvement in convergence rate due to introducing mobility among the leaders.

Experiments in Reference [4,31] demonstrate that making leaders mobile improves the connectivity of the network. As a result, the system reaches a consensus in less number of iterations. For example, Figure 4 shows a scenario in which two leaders are mobile. As a result, the system reaches consensus in six transitions while it takes sixteen iterations to reach consensus if the system is stationary, as shown in Figure 5. In this example, every time S2 and S4 switch their locations, it is considered as a transition. In each iteration, two transitions take place, that is, S2 and S4, exchange their locations, and come back to their original locations. In Figure 5, the number of transitions are compared between stationary and mobile scenarios.

According to Equation (15), a consensus is reached when the product of instantaneous transition matrices converges. In a stationary system, the instantaneous transition matrix is fixed, and the product of the *n* number of the instantaneous transition matrices is $P^{n}$. On the other hand, in a mobile system, the instantaneous system matrix changes according to the dynamics of the network topology. Consensus is said to be achieved when the transition matrix satisfies ( See Equation (15)). In a network, which follows a deterministic mobility model, a *product matrix* is the product of all the instantaneous transition matrices. The characteristics of the product matrix corresponding to a static network are explained in Reference [3]. Theorem 1 explains the characteristics of the product transition matrix corresponding to a mobile network.

### 4.1. Characteristics of the Product Matrix

**Theorem** **1.** *The eigenvalues of the product matrix lie between zero and one. At least one of the eigenvalues is equal to one. If the eigenvalue is complex, its conjugate is also an eigenvalue of the system.*

**Proof of Theorem** **1.** Since the transition matrix is stochastic, the product of stochastic matrices also remains stochastic. As a consequence, one of the eigenvalues of the product of transition matrices is equal to one. The associated eigenvector for this eigenvalue is $1_{n\times 1}$. The eigenvalues associated with the product matrix are real or complex conjugate. The complex eigenvalues exist as the matrix is not symmetric. However, as all the values in each of the instantaneous transition matrices in each state are real, and the product of real numbers remains real, the characteristic equation corresponding to the product matrix will only have real coefficients. As a result, the complex conjugate pairs always appear together as eigenvalues. □

### 4.2. Impact of Mobility on a Disconnected Graph

This section begins with an example to demonstrate that mobility accelerates the convergence rate of consensus in a leader-follower model even in a disconnected network [4,31]. Following this discussion, Theorem 2 proves this point for the class of networks with isolated agents. A disconnected network refers to a network with more than one communication class.

#### Impact of Mobility on a Network with an Isolated Node

**Figure 6:**
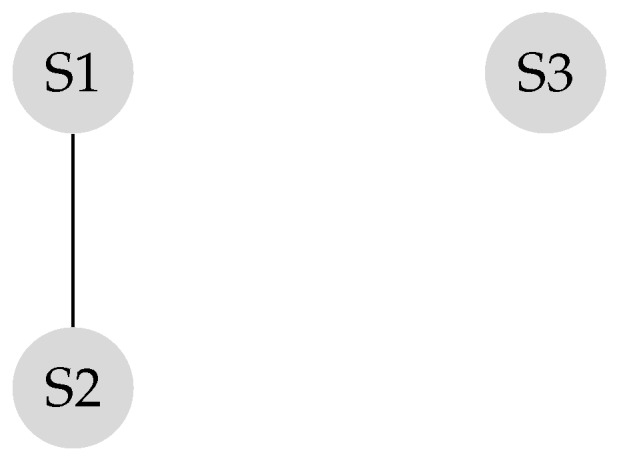
Disconnected graph with an isolated node.

Figure 6 shows an example of a disconnected network with an isolated agent. In this network, sub-network of $\left[S_{1},S_{2}\right]$ represents a communication class, and $\left[S_{3}\right]$ presents another communication class. Consequently, in a graph with stationary leaders, information from an isolated agent ($S_{3}$) remains with this agent. It does not mix with the opinions of the other agents in the sub-network $\left[S_{1},S_{2}\right]$. As a result of having two communication classes, the SLEM of the system equals to one. The set of eigenvalues of the system matrix corresponding to this network is λ={1,1,0}.

As mentioned earlier, the SLEM for this example is one, which means the graph will not reach a consensus. The corresponding transition matrix, *P*, also suggests that increasing the number of iterations will not lead to a consensus.

$$P=\left[\begin{array}{ccc} \frac{1}{2} & \frac{1}{2} & 0\\ \frac{1}{2} & \frac{1}{2} & 0\\ 0 & 0 & 1 \end{array}\right]$$

As illustrated in the above example, sensor S3 is isolated. It is not connected to the rest of the network. Consequently, $p_{33}$ in the transition matrix is one, and the other values in the third row are zeros. As a result of the sub-network associated with S1-S2 and lack of connectivity between S1-S2 and S3, the third column also has zero in every row except for the third row. It breaks the transition matrix into two blocks associated with each communication class. Such a transition matrix will never reach a consensus.

**Figure 7:**
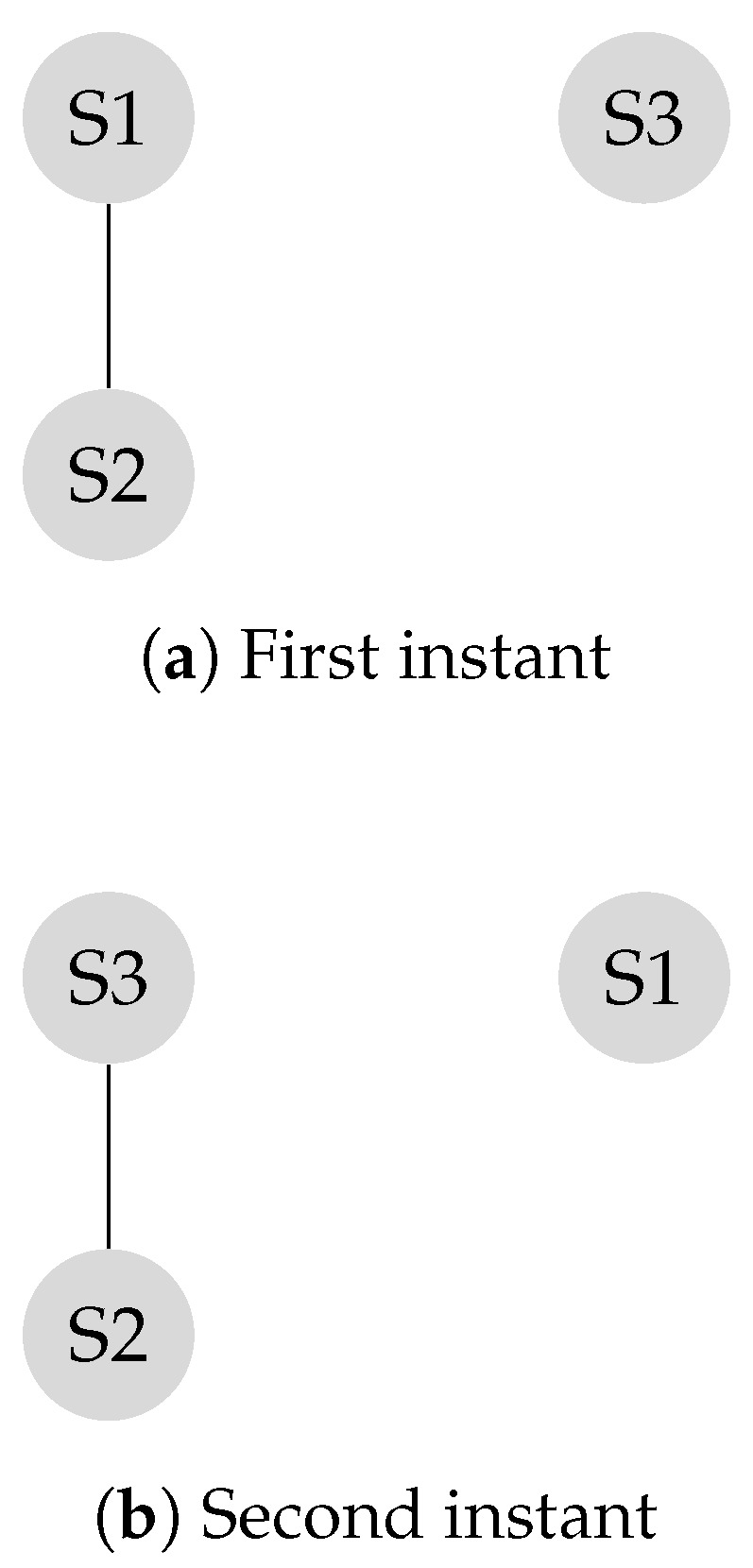
Disconnected graph with mobile leaders.

In order to apply the leader-follower model, the sub-network of S1-S2 needs to be represented as a bipartite structure. The sub-network is made of only two nodes that share the same characteristics. Either of these two nodes can serve as a leader. The isolated node S3 will serve as a leader by itself. Suppose S1 and node S3 are selected to serve as leaders, following a Round Robin mobility model, leaders swap their locations after each iteration as illustrated in Figure 7.

The two instantaneous transition matrices corresponding to the two states are given by $P_{1}$ and $P_{2}$, respectively.

$$P_{1}=\left[\begin{array}{ccc} \frac{1}{2} & \frac{1}{2} & 0\\ \frac{1}{2} & \frac{1}{2} & 0\\ 0 & 0 & 1 \end{array}\right]$$

$$P_{2}=\left[\begin{array}{ccc} 1 & 0 & 0\\ 0 & \frac{1}{2} & \frac{1}{2}\\ 0 & \frac{1}{2} & \frac{1}{2} \end{array}\right]$$

Mobility helps the disconnected network become connected and thus helps the network to reach consensus after a few iterations. The final transition matrix is given by $P_{s}$.

$$P_{s}=\left[\begin{array}{ccc} \frac{1}{3} & \frac{1}{3} & \frac{1}{3}\\ \frac{1}{3} & \frac{1}{3} & \frac{1}{3}\\ \frac{1}{3} & \frac{1}{3} & \frac{1}{3} \end{array}\right]$$

The above example demonstrates that mobility makes consensus possible in a disconnected graph by bringing the disconnected nodes together and facilitating the connectivity. The second theorem proves this hypothesis analytically.

**Theorem** **2.** *Mobility of leader agents enables a network with an isolated agent to reach consensus.*

**Proof of Theorem** **2.** The transition matrix, which was generated using (See Equation (14)), is a square non-negative matrix with positive diagonal elements. For every set of square non-negative matrices with positive diagonal elements the Equation (20) is true [22].

(20) $$P_{1}P_{2}...P_{m}\geq {\left(\frac{\mu^{2}}{2\rho }\right)}^{m-1}\left(P_{1}+P_{2}+...+P_{m}\right).$$

In Equation (20), μ and ρ represent the smallest and the largest elements on the diagonal of the transition matrices $P_{1}$, $P_{2}$, …, $P_{m}$.

To simplify the right side of this inequality, we need to prove Equation (21), that is,

(21) $$P_{1}+P_{2}+...+P_{m}\geq P_{union}.$$

In Equation (21), $P_{union}$ is the transition matrix corresponding to the union of all the instantaneous network typologies. For Equation (21) to be true, for every connection in every instantaneous network topology, the sum of weights of that connection must be equal to or greater than the weight of the corresponding connection in the network of the union (See Equation (22)), that is,

(22) $$p_{ij}\geq p_{\mathrm{union}\;ij}.$$

In order to prove Equation (22), we need to look into two scenarios.

**Scenario 1**: If the *i*th node is a leader, *N* which indicates the number of leaders it has in common with any *j*th node will remain the same (equal to 1) for all the instantaneous matrices. It remains the same in the matrix of the union, as well. If *j* is another leader, then, $p_{ij}$ will stay the same, equal to zero in both $P_{instantaneous}$ and $P_{union}$ matrices because there is no leader common between *i* and *j* as both are leaders.

On the other hand, if *i* is a leader and *j* is a follower, then the value of $p_{ij}$ in the matrix $P_{union}$ will change only if the degree of *i* changes.

Given that the degree of a node in the union graph is equal to or greater than any instantaneous graph, $p_{\mathrm{union}\;ij}$ in the matrix $P_{union}$ is equal to or smaller than $p_{ij}$ in any instantaneous matrix $P_{instantaneous}$.

**Scenario 2**: Consider the case in which *i* is a follower, and *j* is a leader. When the network topology changes due to mobility, either *N* or *c* might increase. In this scenario, $p_{\mathrm{union}\;ij}$ in the union matrix is equal to or smaller than $p_{ij}$ in any instantaneous matrix $P_{instantaneous}$. The value of *M* will not change in this case because the only common leader between a leader and its follower is the leader itself.

Finally, if both *i* and *j* are followers, a different scenario is likely in which the number of mutual leaders between the two followers increases. In this case, there is at least one instantaneous graph with connections between *i*, *j* through leader *k*. Consequently, the $p_{ij}$ in the matrix $P_{instantaneous}$ in the summation of the instantaneous matrices will be equal or greater than $p_{\mathrm{union}\;ij}$ in the matrix of union $P_{union}$. Equation (23) proves the inequality shown in Equation (22), in the scenario in which the number of leaders connected to *i* is not changing, but, the number of common connections between the two nodes *i* and *j* are increasing.

(23) $$\begin{array}{cc} \frac{1}{N}\Sigma_{m=1}^{M}\left(\frac{1}{c_{m}+1}\right)+\frac{1}{N}\Sigma_{m=1}^{M}\left(\frac{1}{c_{m}+1}\right)+\frac{1}{c_{k}+1} & \geq \\ \frac{1}{N}\Sigma_{m=1}^{M}\left(\frac{1}{c_{m}+1}\right)+\frac{1}{c_{k}+1+1} & . \end{array}$$

In Equation (23), *k* is a new leader introduced to follower *i* due to mobility and $c_{k}$ is the degree of *k*.

For any other case involving either a change in *N* or in *c*, the same proof can be used to confirm the inequality ( See Equation (23)). With both scenarios confirming the inequality shown in Equation (21), consequently (20) can be simplified as:

(24) $$P_{1}P_{2}...P_{m}\geq {\left(\frac{\mu^{2}}{2\rho }\right)}^{m-1}\left(P_{union}\right).$$

In Equation (24), the product of the instantaneous transition matrices is greater than or equal to the matrix of the union. The matrix of the union is a modified bipartite graph and hence is aperiodic. The union matrix has a path from every node to every other node. It implies that this matrix is irreducible. The union matrix being aperiodic and irreducible is also primitive [32]. The product matrix is bigger than the union matrix. Hence, the product matrix is primitive, as well [22]. Further, the product matrix is a product of a set of stochastic matrices, so it is stochastic. A stochastic and primitive matrix is ergodic, which means it reaches a consensus if the number of terms in the product is large enough. □

**Figure 8:**
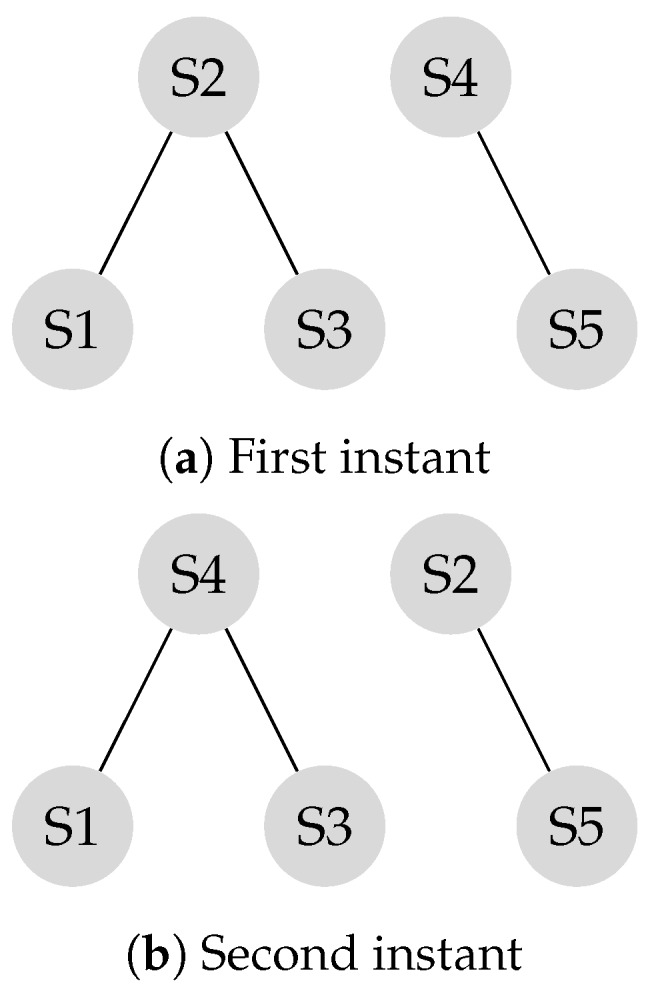
Disconnected graph with mobile leaders.

Theorem 2 discussed the impact of mobility in a leader-follower structure with isolated nodes. A leader-follower model might have multiple communication classes with no isolated node. Figure 8 illustrates a graph with two communications classes and no isolated node.

As illustrated in the example in (Figure 8a), the sub-network of sensors S4-S5 and the sub-network of sensors S1-S2-S3 are disconnected. The corresponding transition matrix is derived according to Equation (14).

$$P_{1}=\left[\begin{array}{ccccc} \frac{1}{3} & \frac{1}{3} & \frac{1}{3} & 0 & 0\\ \frac{1}{3} & \frac{1}{3} & \frac{1}{3} & 0 & 0\\ \frac{1}{3} & \frac{1}{3} & \frac{1}{3} & 0 & 0\\ 0 & 0 & 0 & \frac{1}{2} & \frac{1}{2}\\ 0 & 0 & 0 & \frac{1}{2} & \frac{1}{2} \end{array}\right]$$

Figure 8b represents the network when the mobile leaders of $S_{2}$ and $S_{4}$ switch their locations. Leaders were selected according to the principles of leader selection, which were discussed earlier in this work. The SLEM for this network equals one.

Mobile leaders improve network connectivity, and this network reaches a consensus in a few iterations. The final transition matrix is given by $P_{s}$,

$$P_{s}=\left[\begin{array}{ccccc} \frac{1}{5} & \frac{1}{5} & \frac{1}{5} & \frac{1}{5} & \frac{1}{5}\\ \frac{1}{5} & \frac{1}{5} & \frac{1}{5} & \frac{1}{5} & \frac{1}{5}\\ \frac{1}{5} & \frac{1}{5} & \frac{1}{5} & \frac{1}{5} & \frac{1}{5}\\ \frac{1}{5} & \frac{1}{5} & \frac{1}{5} & \frac{1}{5} & \frac{1}{5}\\ \frac{1}{5} & \frac{1}{5} & \frac{1}{5} & \frac{1}{5} & \frac{1}{5} \end{array}\right]$$

Theorem 3 provides analytical reasoning to confirm the advantages of having mobile leaders in the network with multiple communication classes.

**Theorem** **3.** *Mobility of leaders enables a network with multiple isolated agents or multiple sub-networks reach a consensus.*

**Proof of Theorem** **3.** The proof is similar to that of the Theorem 2. □

Theorems 2 and 3 confirm that in a network with multiple communications classes, introducing mobility to the leaders enables the network reach a consensus.

## 5. Summary and Conclusions

The primary goal of this paper is to study the impact of having mobile nodes in a leader-follower structure. To achieve this goal, it provides theoretical proof in addition to simulation to support the advantages of having mobile nodes in a leader-follower model. This paper analyzes two concepts—leadership and mobility in wireless sensor networks. A strategy based on a bipartite graph is presented to analyze the leader-follower model. An approach based on graph coloring is presented to convert a unipartite graph into a bipartite. A leader selection process is introduced, and the impact of appropriate selection of leaders is demonstrated. The transition matrix associated with consensus-building is discussed in detail. Mobility is explored as a solution for enhancing network connectivity. Finally, this paper provides concrete proofs to demonstrate the benefits of introducing mobility in a sensor network in terms of improved convergence rate in consensus-building.

## Abbreviations

The following abbreviations are used in this manuscript:

SLEM	Second Largest Eigen Module
